# The Effect of Glass Ionomer and Adhesive Cements on Substance P Expression in Human Dental Pulp

**DOI:** 10.4317/medoral.19111

**Published:** 2013-05-31

**Authors:** Javier Caviedes-Bucheli, German Ariza-Garcia, Patricia Camelo, Monica Mejia, Karyn Ojeda, Maria M. Azuero-Holguin, Dunia Abad-Coronel, Hugo R. Munoz

**Affiliations:** 1 Endodontics Department, School of Dentistry, Pontificia Universidad Javeriana, Bogotá, Colombia; 2 Oral Rehabilitation Department, School of Dentistry, Pontificia Universidad Javeriana, Bogotá, Colombia; 3 Postgraduate Endodontics Department, School of Dentistry, Universidad de Cuenca, Ecuador; 4 Postgraduate Endodontics Department, School of Dentistry, Universidad de San Carlos de Guatemala, Guatemala

## Abstract

Objectives: The purpose of this study was to quantify the effect of glass ionomer and adhesive cements on SP expression in healthy human dental pulp. Study Design: Forty pulp samples were obtained from healthy premolars where extraction was indicated for orthodontic reasons. In thirty of these premolars a Class V cavity preparation was performed and teeth were equally divided in three groups: Experimental Group I: Glass Ionomer cement was placed in the cavity. Experimental Group II: Adhesive Cement was placed in the cavity. Positive control group: Class V cavities only. The remaining ten healthy premolars where extracted without treatment and served as a negative control group. All pulp samples were processed and SP was measured by radioimmunoassay. Results: Greater SP expression was found in the adhesive cement group, followed by the glass ionomer and the positive control groups. The lower SP values were for the negative control group. ANOVA showed statistically significant differences between groups (p<0.0001). Tukey HSD post hoc tests showed statistically significant differences in SP expression between negative control group and the 3 other groups (p<0.01). Differences between the cavity-only group and the two experimental groups were also statistically significant (p<0.05 and p<0.01 respectively). There is also a statistically significant difference between the two experimental groups (p<0.01). Conclusions: These findings suggest that adhesive cements provoke a greater SP expression when compared with glass ionomer.

** Key words:**Glass Ionomer, adhesive cement, Substance P, human dental pulp.

## Introduction

Cementing agents are necessary to maintain indirect restorations in place and to fill up the tooth-restoration interphase (1). Continuous development of dental products has provided a wide range of biomaterials to deal with various clinical conditions in dentistry. However, despite these improvements, there is still a need for a biomaterial that completely fulfills all the requirements for a cementing agent, including high biocompatibility, antimicrobial effect, good mechanical properties and to generate appropriate pulpal response (2).

Luting agents used for cementation of crowns, inlays and onlays have been classified in five major categories: a) zinc-oxide and eugenol based cements; b) zinc phosphate cement; c) zinc polycarboxylate cement; d) glass ionomer cements; and e) resin based cements. The first three are no longer used due to their poor mechanical and biological properties (3).

Glass ionomer cements are one of the most used cementing agents in dentistry. However, they have a relatively slow setting, making them highly sensitive to moisture, which decreases their resistance and durability (4). Resin cements are now being widely used, claiming to have better mechanical properties than other cements. Most of these adhesive systems are composed of methacrylate monomers such as hydroxyethyl methacrylate (HEMA), which turn into solid compounds through the polymerization process. However, just 50-75% of the monomers polymerize, the rest remain as free radicals, mainly reactive oxygen species (ROS), which have the ability to diffuse through dentinal tubules constituting a biological risk to the pulp as there is a link between ROS production and cytotoxic activity (5). When pulp fibroblasts are exposed to an increased ROS concentration, they undergo oxidative stress, due to lipids, proteins and DNA alterations, decreasing the mitochondrial activity and cell viability (6). It also has been shown that monomer release can generate a chronic inflammatory response, dentin resorption and immunosuppression (7).

The biological effect of luting cements seems to be controversial in the literature, as there are reports that postoperative sensitivity is a common consequence with the use of resin-based cements, while others report excellent biocompatibility when the cavity is not very deep (2,3). Moreover, an in vivo experiment showed that resin modified glass ionomer cements applied in deep class V cavities generate mild to moderate inflammatory reactions in short (3 to 7 days) experimental periods (8). Postoperative sensitivity after placing glass ionomer cements seems to be related to their low (lower than 3) initial pH (4).

Hyperalgesia and sensitization of nerve fibers are some of the conditions that pulp tissue undergoes when neurogenic inflammation takes place; causing a reduction in pain threshold, increased inflammatory response due to the arrival of vasoactive substances, extravasation of fluids and plasma proteins to the interstitial tissue, and thereby increasing pulp pressure in the injury site (9).

Previous findings have demonstrated how Substance P (SP) is a key neuropeptide in the generation of neurogenic inflammation, as it is increased during caries and inflammation (10), occlusal trauma (11), after cavity preparation (12) and dentin-bonding agents application (13), as well as after the application of tooth bleaching products (14). However, little is known about SP behavior in response to the application of luting cements over dentin. Previous research has focused on describing cellular and histological inflammatory changes in pulp produced by these cements (2,7,8) but there is no evidence to date on the effect of these cements over SP release.

Therefore, the purpose of this study was to determine the effect of the application of glass ionomer cement versus a resin-based cement on SP release in healthy human dental pulp. This knowledge could be useful for assessing SP behavior when routine restorative procedures are carried out, and consequently, contribute to clinician’s decision making to minimize pulp tissue injury.

## Material and Methods

A descriptive comparative study was performed according to Colombian Ministry of Health recommendations regarding ethical issues in research involving human tissue. Written informed consent was obtained from each patient participating in the study. Forty pulp samples were obtained from healthy, nonsmoking human donors (18–30 years old). All teeth used were caries-and restoration-free with complete root development determined both visually and radiographically and without signs of periodontal disease.

Teeth were anesthetized by 1.8 mL 4% prilocaine without vasoconstrictor infiltration injection for maxillary premolars and by inferior alveolar nerve block injection for mandibular premolars. Teeth were divided into four groups containing five maxillary and five mandibular premolars each: (i) Intact-teeth control group: healthy premolars where normal SP values were measured; (ii) cavity-only control group: a Class V cavity preparation was performed; (iii) Experimental group I: a Class V cavity preparation was performed and coated with a glass ionomer cement (Vitremer Cem, 3M Espe, Neuss, Germany); (iv) Experimental group II: a Class V cavity preparation was performed and coated with a resin-based cement (Rely-X Unicem, 3M Espe).

-Experimental procedure and sample collection

For the intact-teeth control group, extraction was performed by conventional methods ten minutes after anesthetic application. For the rest of the groups, a Class V cavity was performed 5 min after anesthetic application. Cavity dimensions were standardized at 1.5 mm height, 3 mm width and 2 mm depth. A cavity was made 1 mm above the cementoenamel junction on the buccal surface of the tooth, with a new cylindrical bur (KG Sorensen No 1093, Sao Paulo, Brazil) marked at 2 mm for cavity depth standardization purposes, with a high-speed handpiece (GentleForce 7000C; Kavo, Biberach, Germany) with abundant irrigation. The bur was used with an intermittent cutting motion until the maximum depth was reached. For the cavity-only control group, teeth were extracted 10 min after the cavity preparation was completed.

For both experimental groups, the luting cements were prepared following manufacturers recommendations and placed in the standardized cavities. Ten minutes later, teeth were extracted by conventional methods.

All teeth were washed with 5.25% sodium hypochlorite after extraction to eliminate remnants of periodontal ligament that could contaminate the pulp sample. The teeth were then sectioned using a Zekrya bur (Dentsply, Tulsa, OK, USA) in a high-speed hand-piece irrigated with saline solution. Remaining dentin thickness was measured after extraction on a proximal digital X-ray of the tooth and directly over the tooth after splitting. Teeth that did not have 1 mm of remaining dentin thickness were discarded. Pulp tissue was obtained using a sterile endodontic excavator, placed on an Eppendorf tube, snap-frozen in liquid nitrogen and kept at -70º C until use.

-Radioimmunoassay (RIA)

Pulp tissue samples were defrosted without thermal shock, dried on a filter and weighed on an analytical balance. Neuropeptide was extracted by adding 150 ?L of 0.5 mol/L acetic acid and double-boiling in a thermostat bath for 30 min in accordance with previously reported protocols (11-15).

SP expression was determined by competition binding assays using a human SP RIA-kit from Phoenix Peptide Pharmaceutical (Ref. RK-061-05, Belmont, CA, USA). The lowest detection limit of this kit is 55 pmol SP per mL.

A total of 50 ?L of each sample solution were incubated in polypropylene tubes at room temperature for 20 h with 100 ?L of primary antibody (1:100 Rabbit anti-SP serum solution) and 100 ?L of different SP concentrations (10 pg/mL –1280 pg/mL). Then, 50 ?L of 125I-SP was added and left incubate for another 24 h. Bound fractions were precipitated by the addition of 100 ?L of a secondary antibody (Goat Anti- Rabbit IgG serum), 100 ?L of normal rabbit serum and 500 ?L of RIA buffer containing 1% polyethylene glycol 4000. After 2 h of incubation at room temperature, tubes were spun at 3000 rpm for 45 min at 4º C. The supernatants were decanted, and pellet radioactivity was read on a Gamma Counter (Gamma Assay LS 5500; Beckman, Fullerton, CA, USA). Standard curves of authentic peptide were made in buffers identical to the tissue extracts on semi log graph paper.

Finally, analysis of the binding data assessed the amount of SP present in every sample, using the percentage of maximum binding (B/B0%) calculated for each unknown sample, reading across the graph to the point of intersection with the calibration curve, where the corresponding X-axis coordinate is equivalent to the concentration of peptide in the assayed sample.

-Statistical analysis

Values are presented as SP concentration in pmol per mg of dental pulp tissue. Mean standard deviation and maximum/minimum values are presented for each group. ANOVA test was performed to establish statistically significant differences between groups (P < 0.05). Tukey HSD post hoc comparisons were also performed.

## Results

SP was found to be present in all pulp samples. Highest SP levels were observed in the Rely-X Unicem group, with a mean value of 2993.77 ± 881.47 pmol SP per mg pulp tissue, followed by the Vitremer Cem group with a mean SP value of 1591.46 ± 316.66 pmol SP per mg pulp tissue. Mean value for the cavity-only control group was 1189.62 ± 205.71 pmol SP per mg pulp tissue. Lowest SP levels were observed in the intact-teeth control group samples with a mean value of 756.94 ± 62.85 pmol SP per mg pulp tissue ( Table 1). ANOVA test showed statistically significant differences between groups (P < 0.0001). Tukey HSD post hoc tests showed significant statistical differences between the intact-teeth control group and the three other groups (P < 0.01). Differences between the cavity-only group and the two experimental groups were also statistically significant (P < 0.05 and P<0.01 respectively). There is also a statistically significant difference between the two experimental groups (P < 0.01).

**Table 1:**
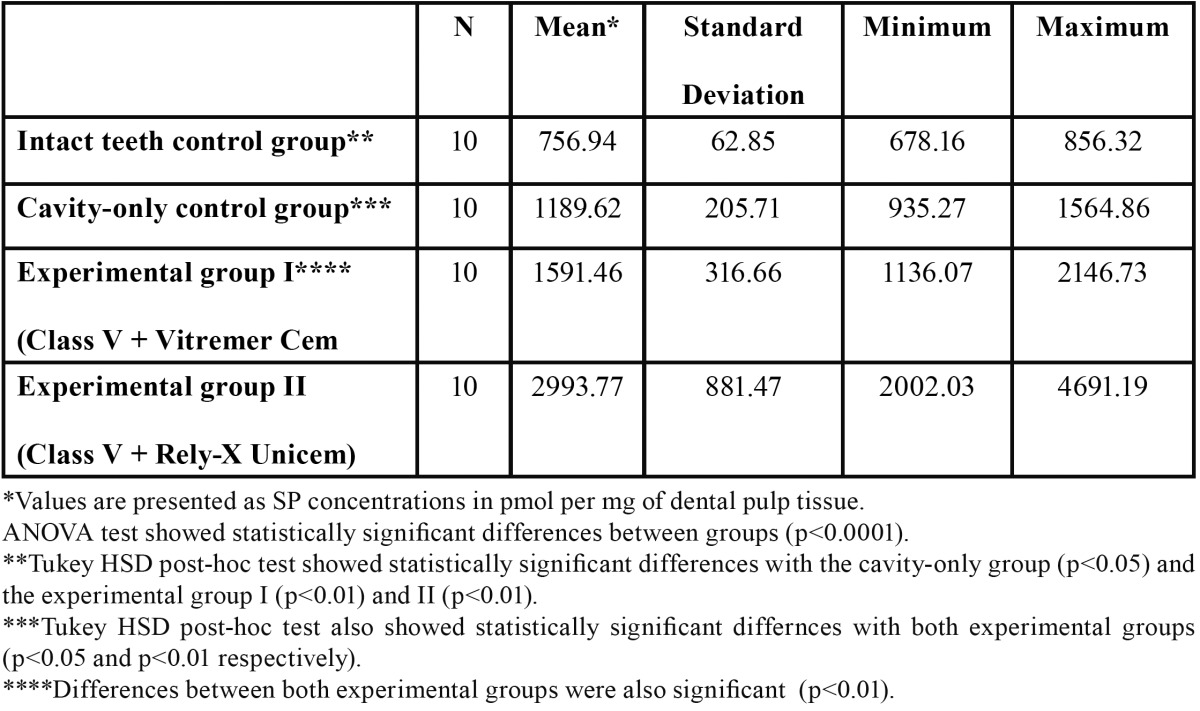
Pulpal SP expression in healthy human premolars after the application of two different cementing agents over Class V cavities.

## Discussion

It has been demonstrated that restorative procedures, such as cavity preparations and placement of restorative materials, constitute physical and chemical irritants to the pulp, because of a rise in pulp temperature, outward movement of dentinal fluid, odontoblast cell-body aspiration and other cytotoxic effects, generating an inflammatory process, that depending on pulp condition may be reversible or irreversible (12,13,16).

The present study found a significant increase in the SP release when cavity preparations were made, which is a finding in accordance with previously published data (12). Cavity preparation induces a pulp reaction that depends on several factors, including mechanical and thermal irritation, injury to the odontoblastic process, remaining dentin thickness and toxicity of subsequent dental materials like luting cements (2,8,16).

Substance P values were obtained from healthy premolars in which extraction was indicated for orthodontic reasons. Local anesthetic used in all groups of this study was 4% prilocaine without vasoconstrictor to prevent neuropeptide expression becoming attenuated by vasoconstrictors as previously demonstrated (15).

Because of the ‘in vivo’ nature of the present study, it was difficult to standardize remaining dentin thickness to a fixed measure. However, the cavity standardization method used has proven to be effective to minimize the variation in remaining dentin thick-ness (13). Cavities were made 1 mm above of cementoenamel junction, where, according to published data (17), dentin thickness is approximately 3 mm. Therefore, with a 2 mm depth cavity, it was expected that the remaining dentin thickness was 1 mm. This was confirmed measuring remaining dentin after extraction on a proximal X-ray of the tooth and directly over the tooth after splitting. Teeth that didn’t meet this criterion were discarded.

Previous studies have demonstrated that remaining dentin thickness greater than 0.5 mm has a protective effect against external injury factors, such as dentinal adhesives (18). When remaining dentin thickness is less than 0.5 mm, monomers from resins have been observed in the odontoblastic layer and the predentin zone; producing a foreign body reaction, characterized by the presence of an inflammatory infiltrate of macrophages and multinucleated giant cells (19). Moreover, monomers affect the mitochondrial activity in fibroblasts and the secretion of inflammatory mediators in macrophages (7), they also inhibit the proliferation of T lymphocytes and interrupt cell cycle of pulpal cells inducing apoptosis (20). Therefore, no tooth with less or more than 1 mm remaining dentin thickness was used.

In the present study, there was a 10 min delay after application of the cement before proceeding with tooth extraction. As SP release is immediate, calcium-dependent and of short-term, this period of time appears to be sufficient for allowing the neuropeptide to be released from terminal fibers, prior to being degraded by endogenous peptidases (21). It has been speculated on the possible mechanisms for the increase in extracellular SP, including: (i) increased synthesis of the neuropeptide in the trigeminal ganglia; (ii) increased rate of transport; (iii) increased release; and (iv) decreased levels of peptidases, which would result in decreased degradation of SP (22). More recent evidence has demonstrated that mRNA transcripts are transported to peripheral terminals, suggesting that peptide synthesis could occur directly in the peripheral terminals (23). However, in the present study, it is assumed that increased SP levels are more related to increased release, as 10 min would not be enough for the other hypothesis to take place.

Resin-based cements generated a significantly greater SP release compared to the cavity-only and the glass ionomer groups. This could be explained by the ability of HEMA monomers to diffuse through dentine because of its low molecular weight and hydro-philic characteristics (24). These monomers affect the underlying odontoblastic layer, altering cell cycle and generating inflammatory changes in pulp (25). The highly hydrophilic feature of the monomers also increases the permeability of the hybrid layer, thus the penetration of humidity along with the continuous movement of dentinal fluids, interferes with the polymerization rate of the adhesive, increasing the amount of methacrylate ions that could penetrate the pulpodentin complex (26).

It has been shown that the diffusion of HEMA will take place during the initial 15–30 s of polymerization reaction with the high-est monomer liberation in the first 4 min after application and curing, decreasing up to 24–72 h later (27). Some concerns arise regarding clinical meaning of this immediate increase in SP release after application of resin-based cements. Measure of pulpal SP release in the present research was carried out in the time interval where the highest HEMA liberation was expected. However, it cannot be assured that SP levels will behave in the same way on the long-term, as previous studies have found that release of methacrylate ions continues for as long as 30 days after restoration placement (28). Therefore, it is probable that in the long-term, SP expression could be affected by long-standing monomer release and, because of the free radical effects on pulp tissue (i.e. cell apoptosis), it is possible to trigger a chronic inflammatory response, which may reduce pulp ability to defend itself from future injuries, therefore gaining clinical significance (27).

On the other hand, the slow setting of glass ionomer cement involves an acid-base reaction, making it highly sensitive to humidity. This slow setting constitutes a considerable aggression to the pulp, while the chemical reaction and the pH of the cement be-come stable (29). This could be the reason why SP levels were significantly higher in the glass ionomer cement group when compared to the cavity-only group. However, this increase was not as high as the resin-based group. This could be explained by the smaller particle size of the components of resin-based cements, such as HEMA, allowing an easier diffusion of this monomer through dentinal tubules (24).

This study proves that restorative procedures, such as Class V cavity preparations, produce a significant increase in the SP levels in human dental pulp compared with baseline levels of this neuropeptide. Application of luting cements significantly increases SP release in dental pulp; with a statistically significant difference between the increase provoked by a resin-based cement when compared to glass ionomer cement. It is important to point out that these increases in SP alone may not have any clinical relevance by itself. However, biologic effects of cumulative injuries to pulpodentin complex should not be considered separately, as consequences derived from one factor are virtually impossible to separate from the combined effects of all other factors (30). Clinicians should be aware that, performing a restorative procedure even under the best conditions, represents an offense to the pulpodentin complex, which could generate several neurogenic and vascular reactions, including the release of SP.
